# Supplementary material for: Oxidation of Archean upper mantle caused by crustal recycling
Source: Nat Commun. 2022 Jun 7;13:3283. doi: 10.1038/s41467-022-30886-4 (PMC9174474; doi:10.1038/s41467-022-30886-4)
Supplement: Supplementary file 1 — Supplementary Information [file 41467_2022_30886_MOESM1_ESM.pdf]

Supplementary Information for

Oxidation of Archean upper mantle caused by crustal recycling

Lei Gao[1,2], Shuwen Liu[2*], Peter A. Cawood[3*], Fangyang Hu[4,5], Jintuan Wang[6], Guozheng Sun[2], Yalu Hu[2]

[1] *Key Laboratory of Geological Processes and Mineral Resources, School of Earth Sciences and Resources, China University of Geosciences, Beijing 100083, PR China*

[2] *Key Laboratory of Orogenic Belts and Crustal Evolution, Ministry of Education, School of Earth and Space Sciences, Peking University, Beijing 100871, PR China*

[3] *School of Earth, Atmosphere and Environment, Monash University, Melbourne, VIC 3800, Australia*

[4] *Key Laboratory of Mineral Resources, Institute of Geology and Geophysics, Chinese Academy of Sciences, Beijing 100029, China*

[5] *Innovation Academy for Earth Science, Chinese Academy of Sciences, Beijing 100029, China*

[6] *State Key Laboratory of Isotope Geochemistry, Guangzhou Institute of Geochemistry, CAS, Guangzhou 510640, PR China*

***corresponding authors**

Shuwen Liu (swliu@pku.edu.cn)

Peter A. Cawood (peter.cawood@monash.edu)

Supplementary Figures

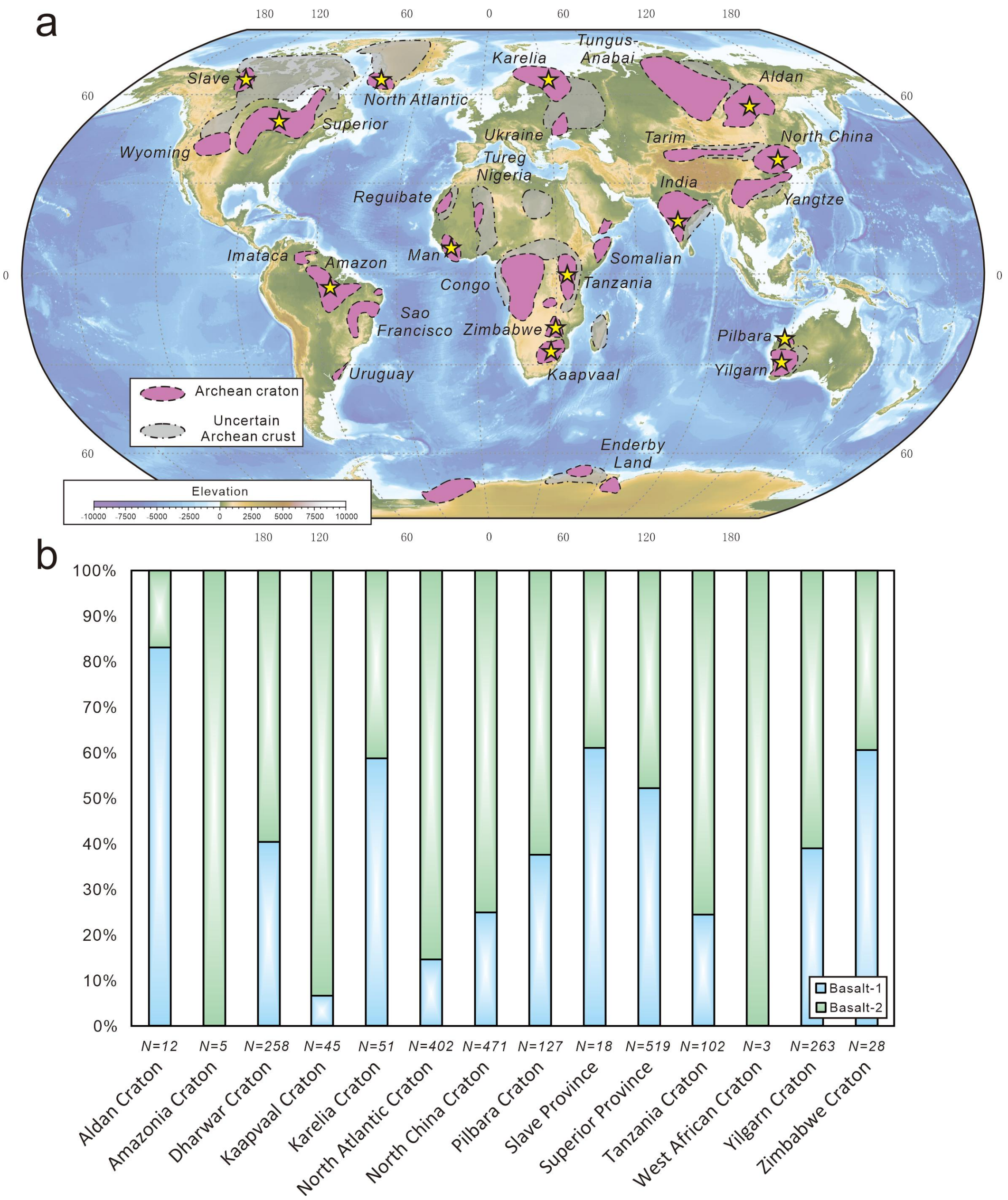

Supplementary Figure 1 Sample distribution of Archean basalts in global cratons. a, Map of exposed Archean cratons. This world map is created using the Generic Mapping Tools v6.3.0 (https://iso.mirrors.ustc.edu.cn/gmt/bin/gmt-6.3.0-win64.exe). The outline of Archean cratons is modified from Condie et al[1]. According to statistics, at least fourteen cratons have reliable basalt records. **b,** Percentage stacking bar chart of the Archean basalts in respective cratons.

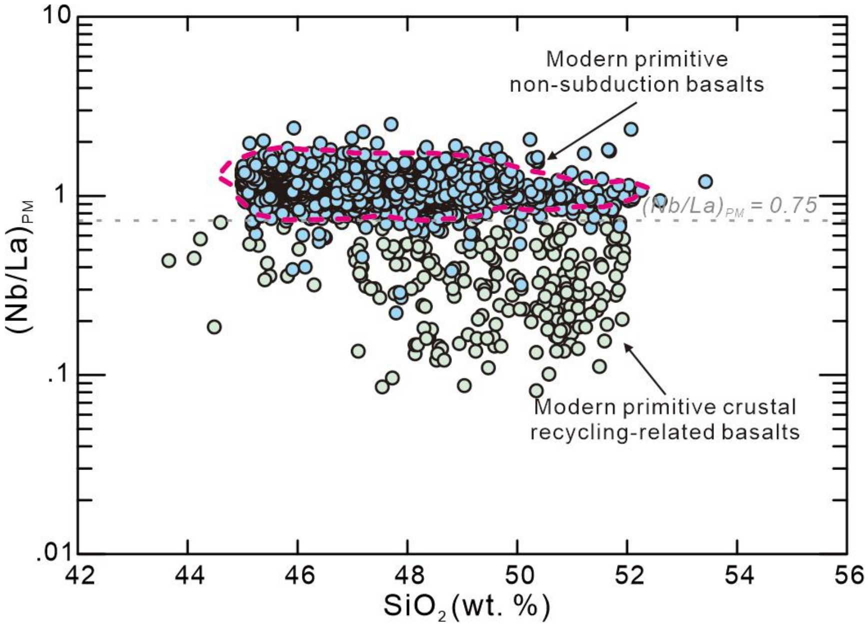

Supplementary Figure 2 Geochemical discrimination of modern non-subduction and subduction basalts. The outlined area was the compositions of modern samples collected from mid-oceanic ridges, oceanic islands and oceanic plateaus, of which parental magmas were not metasomatized by slab-derived fluids or melts and carbonatitic melts (data from the GEOROC database; http://georoc.mpch-mainz.gwdg.de/georoc/). Through a kernal density statistics of modern basalts with definite tectonic settings, we found that $(Nb/La)_{PM} \geq 0.75$ is a crucial identification of the non-subduction basaltic melts, distinguishing from the primitive melts that generated at subduction-related settings with $(Nb/La)_{PM} < 0.75$[2].

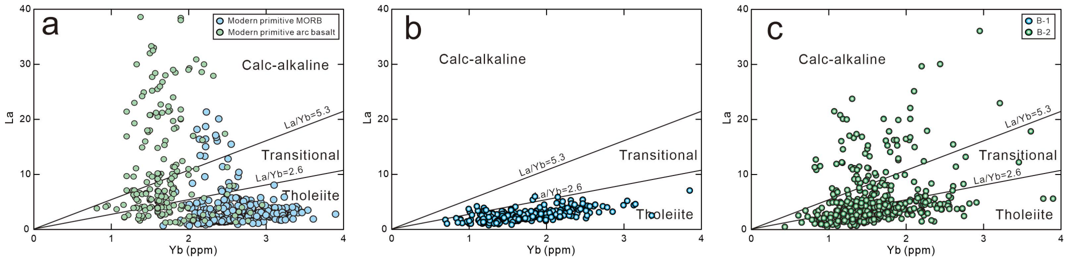

Supplementary Figure 3 La versus Yb geochemical classification diagrams for basalt samples. a, For modern primitive mid-ocean ridge basalts (MORBs) and arc basalts, respectively[3]. The modern primitive MORBs are from the GEOROC database (http://georoc.mpch-mainz.gwdg.de/georoc/), and the primitive arc basalts are from Schmidt and Grunder[2]. **b,** For Basalt-1 (B-1) samples. **c,** For Basalt-2 (B-2) samples.

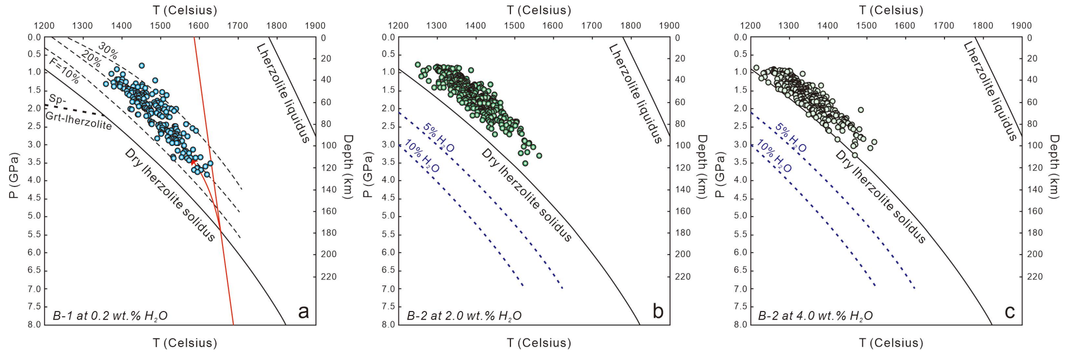

Supplementary Figure 4 Temperatures and pressures calculated for primary magmas of Archean basalts. a, b and c, Calculated results for Basalt-1 (B-1) and Basalt-2 (B-2) samples (2 and 4 wt.% H_2O), respectively. Lherzolite solidus and melt fraction isopleths are from Katz et al[4]. The tendency of phase transition between spinel and garnet mantle peridotite is from O'Neill[5]. The potential temperature (T_P) values were estimated by back calculating the melting conditions of the primary magmas along an isentropic melting adiabat until the melting adiabat intersected the solidus and then extrapolating from this intersection point along a solid mantle adiabat to the surface[6]. The red curved line with an arrow corresponds to the isentropic melting adiabat, and the near-vertical red lines represent the solid mantle adiabats with varying T_P.

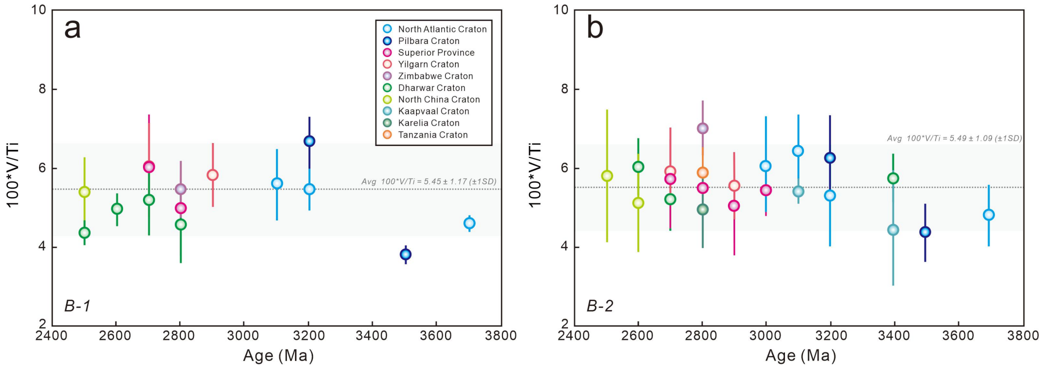

Supplementary Figure 5 Time evolution of V/Ti ratios. a, For Archean Basalt-1 (B-1) samples. **b,** For Archean Basalt-2 (B-2) samples. The average 100*V/Ti values and their uncertainties are shown as grey intervals. Error bars show the 1 standard deviation of the means.

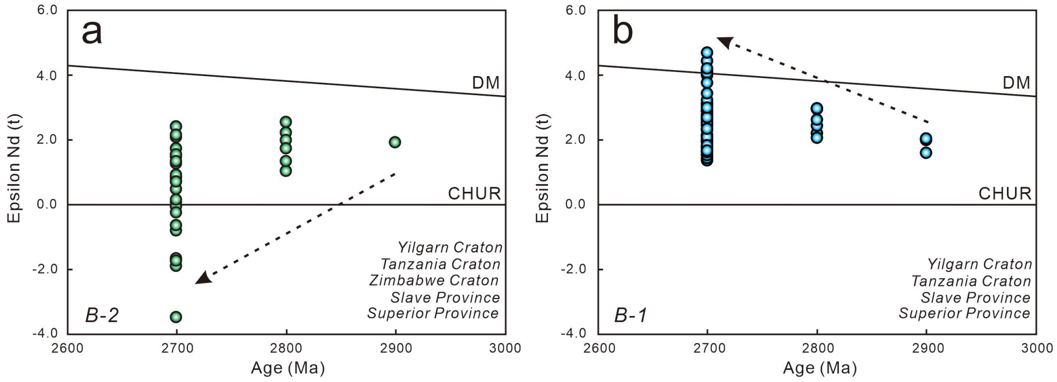

Supplementary Figure 6 Time evolution of Nd isotope ratios. a, For Basalt-2 (B-2) samples from the representative Archean cratons. **b,** For Basalt-1 (B-1) samples from the representative Archean cratons. The Nd isotope compositions are from the GEOROC database (http://georoc.mpch-mainz.gwdg.de/georoc/).

References

1. Condie, K. C., Belousova, E., Griffin, W. L. & Sircombe, K. N. Granitoid events in space and time: Constraints from igneous and detrital zircon age spectra. *Gondwana Res.* **15**, 228-242 (2009).

2. Schmidt, M. E. & Grunder, A. L. The evolution of North Sister: A volcano shaped by extension and ice in the central Oregon Cascade Arc. *Geol. Soc. Am. Bull.* **121**, 643-662 (2009).

3. Ross, P. S. & Bédard, J. H. Magmatic affinity of modern and ancient subalkaline volcanic rocks determined from trace-element discriminant diagrams. Can. *J. Earth. Sci.* **46**, 823-839 (2009).

4. Katz, R.F., Spiegelman, M. & Langmuir, C.H. A new parameterization of hydrous mantle melting. *Geochem. Geophys. Geosyst.* **4**, 1073 (2003).

5. O'Neill, H. S. C. The transition between spinel lherzolite and garnet lherzolite, and its use as a geobarometry. *Contrib. Mineral. Petrol.* **77**, 185-194 (1981).

6. Lee, C.-T. A., Luffi, P., Plank, T., Dalton, H. & Leeman, W. P. Constraints on the depths and temperatures of basaltic magma generation on Earth and other terrestrial planets using new thermobarometers for mafic magmas. *Earth Planet. Sci. Lett.* **279**, 20-33 (2009).
