# Supplementary material for: Oxidation of Archean upper mantle caused by crustal recycling
Source: Nat Commun. 2022 Jun 7;13:3283. doi: 10.1038/s41467-022-30886-4 (PMC9174474; doi:10.1038/s41467-022-30886-4)
Supplement: Supplementary file 3 — Description of Additional Supplementary Files [file 41467_2022_30886_MOESM3_ESM.pdf]

Description of Additional Supplementary Files

File name: Supplementary Data 1

Description: Geochemical database of the~3.8-2.5 Ga basalts from fourteen Archean cratons.

File name: Supplementary Data 2

Description: Macro file of oxygen fugacity calculation.
